# Insights into the Anti-inflammatory and Antiviral Mechanisms of Resveratrol

**DOI:** 10.1155/2022/7138756

**Published:** 2022-08-12

**Authors:** Xiangxiu Chen, Xu Song, Xinghong Zhao, Yu Zhang, Yiming Wang, Renyong Jia, Yuanfeng Zou, Lixia Li, Zhongqiong Yin

**Affiliations:** ^1^Natural Medicine Research Center, College of Veterinary Medicine, Sichuan Agricultural University, Chengdu 611130, China; ^2^Key Laboratory of Animal Disease and Human Health of Sichuan Province, Sichuan Agricultural University, Chengdu 611130, China

## Abstract

Resveratrol is a naturally occurring stilbene phytoalexin phenolic compound, which has been extensively studied on its biological activity. It has been widely accepted that resveratrol possesses anti-inflammatory and antiviral activities. In this review, we summarize the anti-inflammatory dosages and mechanism and antiviral mechanism of resveratrol. Since viral infections are often accompanied by inflammation, we propose that the NF-*κ*B signaling pathway is a key and common molecular mechanism of resveratrol to exert anti-inflammatory and antiviral effects. For future studies, we believe that resveratrol's anti-inflammatory and antiviral mechanisms can consider the upstream signaling molecules of the NF-*κ*B signaling pathway. For resveratrol antivirus, future studies can be conducted on the interaction of resveratrol with key proteins or important enzymes of the virus. In addition, we also think that the clinical application of resveratrol is very important. In short, resveratrol is a promising anti-inflammatory and antiviral drug, and research on it needs to be expanded.

## 1. Introduction

**Figure 1:**
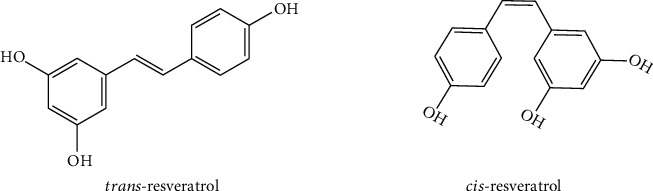
Chemical structures of *trans*- and *cis*-resveratrol (3,5,4′-trihydroxystilbene).

Resveratrol is a natural bioflavonoid compound produced by various members of the family of spermatophytes, such as grapes, mulberry, red wine, and peanuts (summarized in Table 1) [1, 2]. The chemical structure of resveratrol is consisting of two phenolic rings which are joined by a double styrene bond, thus forming the 3,5,4′-trihydroxystilbene with a molecular of weight 228.25 g/mol (Figure 1) [3, 4]. Resveratrol exists in two forms, *trans* and *cis*, mainly dominating is trans. The trans-isomer is more abundant and biologically active than the cis-isomer. However, resveratrol is insoluble in water, but soluble in ethanol and dimethyl sulfoxide and other polar solvents. Therefore, the application of resveratrol has caused some difficulties [5]. To overcome these challenges, resveratrol has been developed in various dosage forms, mainly nanoformulations, which increase its solubility and prevent its degradation while maintaining its biological activity [6–8]. In recent years, due to its environmental friendliness and multiple bioactivities, resveratrol has attracted much attention. Among them, resveratrol with anti-inflammatory, antiviral, anticancer, and cardiovascular protection activities has become a research hotspot [9–11]. Due to its enormous beneficial health effects on the treatment of various human diseases, it is expected to become a new drug to prevent and treat diseases. In this review, we summarized the anti-inflammatory and antiviral mechanisms of resveratrol, providing a basis for its further development and utilization.

## 2. The Anti-Inflammatory Activity of Resveratrol

### 2.1. Anti-inflammatory Effects of Resveratrol on Meningitis

Bacterial meningitis (BM) is an infectious disease characterized by infection and inflammation of the meninges with high morbidity and mortality worldwide. BM is a global public health problem [22]. Despite qualified intensive care and effective antibacterial therapy, BM is still associated with high mortality rates and incidence of neurological sequelae, which are mainly due to neuron loss by necrosis in the cerebral cortex and by apoptosis in the hippocampal dentate granule cells [23]. Studies have shown that activation of microglia to produce excessive proinflammatory factors plays an important role in brain injury [24]. These proinflammatory mediators increase the permeability of the blood-brain barrier (BBB), attracting leukocytes into the central nervous system.

Resveratrol had anti-inflammatory and neuroprotective effects and could cross the BBB [25]. Many studies have shown that resveratrol had a beneficial effect on bacterial meningitis. Resveratrol exhibited inflammatory and neuroprotective effects on an infant rat model of pneumococcal meningitis. The results suggested that resveratrol has been shown to target miRNA expression associated with bacterial meningitis and reduce apoptosis index and the expressions of IL-1*β* and CCL3 [24]. It was confirmed *in vitro* that resveratrol attenuated caveolin-1 (CAV-1) upregulation and inhibited ERK1/2-VEGFA signaling cascade. Resveratrol treatment can reduce the expression of chemokines and improve the survival rate of mice with Escherichia Coli-induced meningitis [26].

Inflammation of the central nervous system mainly includes bacterial meningitis, experimental autoimmune encephalomyelitis, and multiple sclerosis. miR-223 plays an important role in the autophagy of microglia in the brain. Resveratrol regulated the miR-223-3p/NLRP3 pathway, inhibited downstream caspase-1 activation and the processing of IL-1*β* and IL-18 in neurons and BV-2 cells, and protected cortical neurons from inflammatory damage and death [27]. Studies suggested that the excessive inflammatory response caused by bacterial meningitis enhances oxidative stress, which in turn influences acute bacterial meningitis- (ABM-) related neuronal dysfunction or cell death formation [28]. In a *Klebsiella pneumonia*-induced ABM model, resveratrol treatment could significantly reduce the levels of calcium strength, microglial activation, proinflammatory cytokines, and MDA. The mechanism is that resveratrol increases the number of hippocampal neurons in the ABM model by inhibiting microglial activation [23].

### 2.2. Effects of Resveratrol on Rheumatoid Arthritis

Rheumatoid arthritis (RA) is a systemic autoimmune disease primarily affecting joints and characterized by abnormal proliferation of fibroblast-like synoviocytes (FLS), destructive cartilage damage, and joint stiffness and deformity [29]. The clinical manifestations of symmetrical joint involvement include arthralgia, swelling, redness, and even limiting the range of motion [29]. The etiology of the disease involves different mechanisms, and the inflammatory cytokines play a major role [30]. Although RA can be relieved by surgery, there is no medicine available to cure it currently. Resveratrol is a potent activator of SIRT1, which regulated inflammation by deacetylating the transcription factor histone [31]. Many studies had shown that resveratrol reduced infiltration of inflammatory cells and inhibited synovial cell proliferation in synovial tissue [32]. In *in vivo* studies, resveratrol ameliorated RA through activation of the Nrf2-ARE signaling pathway via the SIRT1/NF-*κ*B/miR-29a-3p/Keap1 and SIRT1/NF-*κ*B/miR-23a-3p/cul3 signaling pathway [33]. Upregulation of SIRT1 by resveratrol suppressed the BK-induced COX-2/PGE2 production through inhibiting the interaction of AP-1 and NF-*κ*B with COX-2 promoter in rheumatoid arthritis synovial fibroblasts [34].

Moreover, TNF-*α*, as a proinflammatory factor, is an inflammatory cascade center that regulates immune response, modulates cellular and humoral immunity, and induces the activation of macrophages and osteoclasts, leading to the occurrence and development of synovitis, cartilage erosion, and bone destruction [35, 36]. Interestingly, the PI3K/Akt signaling pathway widely exists in synovial tissues, which can be activated by several cytokines such as TNF-*α* in RA synoviocytes [37]. Studies have shown that resveratrol inhibits TNF-*α*-induced IL-1*β* and MMP-3 production by inhibiting PI3K/Akt signaling in RA FLS [38]. In addition, resveratrol acts as an inhibitor of several signaling pathways in inflammation. The study proved that resveratrol inhibited particulate matter-induced ERK1/2, p38 MAPK, and Akt activation and ROS/NF-*κ*B activation in human FLS [39]. In H_2_O_2_-induced FLS, resveratrol inhibited ROS production by activating the Nrf2 pathway, thereby inhibiting activation of NF-*κ*B and proliferation [40].

### 2.3. Effects of Resveratrol on Pneumonia

Pneumonia is a common respiratory disease in clinic with high morbidity and mortality, especially in the current pandemic COVID-19 [41]. Typically, it is characterized by acute onset, high fever, choking cough with small mucous sputum amounts, chest pain, shortness of breath, and cyanosis, even death due to respiratory distress syndrome [42]. Inflammation is a delicate balancing act. In the pneumonia microenvironment, several factors such as immune cells and biomolecules play a vital role. When the balance of inflammation is disrupted in an infectious environment, the uncontrolled release of immune cells and inflammatory factors leads to intense inflammation in the lungs [43]. It is widely accepted that cytokines, especially IL-10, TNF-*α*, IL-1*β*, IL-6, and IL-8, play an important role in the initiation or execution of lung injury [44]. IL-10 is an important regulator of lung inflammation, which could be an effective adjunct therapy for antibiotics in the treatment of pneumococcal pneumonia [45]. At present, many studies have shown that resveratrol had anti-inflammatory effects [46]. Resveratrol reduced the secretion of cytokines such as TNF-*α*, IL-1*β*, and IL-6 in a model of pneumonia caused by Serratia marcescens infection [47]. NF-*κ*B belongs to a family of inducible nuclear transcription factors that regulated a wide range of genes involved in various processes of inflammation and immune response [48]. The signaling molecules activated NF-*κ*B by degrading IkB, and the activated NF-*κ*B entered the nucleus and bound to DNA, thereby inducing the expression of many inflammatory mediators including TNF-*α*, IL-1*β*, and IL-6 [49]. In human lung epithelial cells, Staphylococcus aureus induces I*κ*B and NF-*κ*B p65 phosphorylation and NF-*κ*B p65 translocation, and resveratrol reduced phosphorylation and NF-*κ*B p65, thereby alleviating pneumonia [50].

## 3. Anti-inflammatory Mechanism of Resveratrol

**Figure 2:**
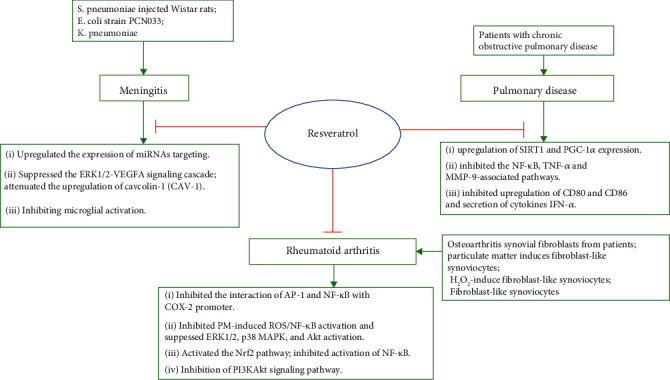
Mechanisms by which resveratrol regulates various inflammatory models.

Resveratrol exhibited strong anti-inflammation properties through several ways (Figure 2). The most widely reported mechanism is that resveratrol could regulate small molecules in multiple signaling pathways, thereby inhibiting the production of inflammatory factors. Human acute respiratory distress syndrome (ARDS) is an inflammatory disorder characterized by a variety of stimuli such as pneumonia, sepsis, trauma, and certain infection [51]. Resveratrol inhibited MDA levels and SOD activity through blocking the ERK and PI3K/Akt pathways in the ARDS model [52]. Moreover, resveratrol treatment reduced the secretion of TNF-*α*, IL-6, and IL-1*β* in the lungs and NR8383 cells by attenuating inflammation via the p38 MAPK/SIRT1 pathway [53]. Myd88-dependent TLR4 and NF-*κ*B pathways are markedly decreased by resveratrol [54, 55]. In the colitis mouse model, resveratrol could ameliorate structural changes in the intestine and reduce the levels of proinflammatory cytokines, including IL-1*β*, IL-6, IL-8, and TJ proteins. Its mechanism of action was that resveratrol inhibited phosphorylation of inflammatory signaling molecule NF-*κ*B, extracellular signal-regulated kinase, and stress C-Jun N terminal protein kinase [56]. It is well known that Toll-like receptors (TLRs) played an important role in the activation of the innate immune system. The TLR signaling pathway was activated by recognizing specific patterns of stimuli, subsequently promoting phosphorylation of the NF-*κ*B signaling pathway, which could increase proinflammatory cytokine secretion, leading to gastrointestinal tract injury [57]. Resveratrol could downregulate the expression level of the TLR4 pathway under inflammatory conditions. From what has been discussed above, resveratrol could regulate the vital TLR/NF-*κ*B pathway of intestinal inflammation to achieve anti-inflammatory effects. In addition, other studies have suggested that the PI3K/Akt/mTOR signaling pathway may be a potential target of inflammation-related diseases. In radiation-induced intestinal injury, resveratrol reduced the levels of inflammatory cytokines by regulating the PI3K/Akt/mTOR pathway [58, 59].

Recently, it was reported that *Helicobacter pylori* infection caused gastric and duodenal inflammation [60]. Meanwhile, studies also have shown that interleukin-8 overexpression and iNOS production could be detected in gastritis models. iNOS is an inflammation-inducing enzyme and an important pathogenic factor in gastritis caused by *H. pylori* [61]. Resveratrol decreases *H. pylori*-induced gastric inflammation by suppressing the proinflammatory mediator IL-8 and the expression of iNOS through the activation of the Nrf2/HO-1 way [62]. In addition, resveratrol was able to mitigate gastritis through increasing the levels of nitric oxide, sialic acid, gastric tissue, and vitamin C concentrations and reducing NF-*κ*B/p65 and proinflammatory cytokines [63].

Overall, several convincing studies have demonstrated that resveratrol had a good role in the prevention and treatment of many inflammatory chronic diseases (Figure 2). However, its molecular mechanism is complex and involves many signal transduction pathways, which have not been fully elucidated.

## 4. The Antiviral Activity of Resveratrol

### 4.1. Effects of Resveratrol on DNA Viruses

#### 4.1.1. Herpes Simplex Virus

The herpes simplex virus (HSV) is a common human, double-stranded DNA virus belonging to the Herpesviridae family. Herpes simplex viruses include types HSV-1 and HSV-2. HSV infection can cause lesions in different parts of the body, including the mouth, eyes, nose, skin, and mucosa. After primary infection of epithelial cells, the virus becomes latent in neurons of the peripheral nervous system and can be periodically reactivated resulting in recurrent clinical or subclinical episodes throughout life [64]. Many studies have shown that resveratrol can inhibit HSV infection *in vivo* and *in vitro*. When added within 1 hour after infection *in vitro*, resveratrol showed potent anti-HSV activity, and the effect decreases and even disappears as time goes on [65]. Resveratrol suppresses HSV through activation of NF-*κ*B within the nucleus in Vero cells and expressions of essential immediate-early, early, and late HSV genes and synthesis of viral DNA [66]. Resveratrol regulated HSV-2 infection by increasing histone acetylation [67].

In an animal study, when treatment was initiated 1 h after HSV infection in mice and repeated 5 times every 3 h for 5 days, both 12.5 and 25% resveratrol cream significantly inhibited the development of HSV-1-induced skin lesions. Animal skin has no apparent dermal toxicity, such as erythema, scaling, crusting, lichenization, or abrasions [68]. Resveratrol is effective not only in skin disease caused by HSV infection but also in vaginal infection. The 19% resveratrol cream administered intravaginally five times a day for 5 days significantly suppressed HSV-2 replication and prevented extravaginally disease [69].

#### 4.1.2. Varicella-Zoster Virus

Varicella-zoster virus (VZV) is a member of the Herpesviridae family. VZV is the causative agent of chickenpox and a common infantile illness. Like all herpesviruses, VZV undergoes a lifelong latent state following primary infection. During latency, the viral DNA persists in the dorsal root ganglia and cranial root ganglia [70]. VZV infection can cause chickenpox and viremia, characterized by fever and watery herpes [71]. Resveratrol reduced VZV replication *in vitro* in a dose- and time-dependent manner. It could completely block the replication of VZV at a concentration of 219 *μ*M within 30 h postinfection. Interestingly, resveratrol does not directly block VZV adhesion or inactivate virion but interferes with the first stage of VZV replication by inhibiting IE62 gene activation and IE62 protein deletion [72]. Additionally, it was reported that oxyresveratrol can inhibit VZV infection with IC_50_ values of 12.82, 12.80, 12.99, and 12.82 *μ*g/mL against wild-type, thymidine kinase-deficient, and two types of DNA polymerase mutants with acyclovir-resistant VZV, respectively [73]. At present, studies on the antiviral activity of resveratrol against VZV mainly focus on the dose of resveratrol, and there are limited reports on the antiviral mechanism.

#### 4.1.3. Pseudorabies Virus

Pseudorabies virus (PRV) is a herpesvirus of swine, a member of the *Alphaherpesvirinae* subfamily, and the causative agent of Aujeszky′s disease (AD) in swine, causing respiratory, neurological, and reproductive symptoms. The infection of PRV in pigs typically starts in the nasal mucosa after viral particles infect sensory neuronal cells. Through retrograde neuronal transport, viral particles travel toward the trigeminal ganglia and olfactory bulb [74]. PRV is always fatal in newborn piglets and often accompanied by neurological symptoms, leading to miscarriage and mummified fetuses in pregnant sows [75]. Due to the emergence of mutant strains, the protection of vaccines has become ineffective. However, several studies have shown that resveratrol has anti-PRV activity. *In vitro*, resveratrol potently suppressed PRV replication in a dose-dependent manner, with IC_50_ of 17.17 *μ*M. The inhibitory effect of resveratrol on PRV-induced cell death and gene expression may be due to its ability to inhibit the degradation of I*κ*B kinase [76]. The NF-*κ*B pathway is known to integrate signaling associated with very diverse intra- and extracellular stressors, including virus infections, and triggers a proinflammatory response through the expression of NF-*κ*B-regulated genes [77]. Indeed, PRV infection continuously activates NF-*κ*B, which regulates the expression of host-related inflammatory factors. Hence, NF-*κ*B may be the key to host resistance to PRV [78]. Additionally, in an animal study, it has been reported that resveratrol can relieve inflammation, reduce pathological changes, and enhance immunity in PRV-infected piglets [79]. PRV infection causes stillbirth and miscarriage, resulting in poor growth of the offspring. Resveratrol treatment alleviates virus-induced reproductive failure and restores serum progesterone levels [80].

### 4.2. Effects of Resveratrol on RNA Viruses

#### 4.2.1. Respiratory Syncytial Virus

Respiratory syncytial virus (RSV) is a filamentous enveloped, negative-sense, and single-stranded RNA virus belonging to the *Orthopneumovirus* genus of the Pneumoviridae family in the order *Mononegavirales* [81]. RSV is responsible for acute respiratory tract diseases, which infect almost all children under 2 years of age. RSV infection can present as a variety of clinical syndromes including upper respiratory tract infections, bronchiolitis, pneumonia, exacerbations of asthma, and viral-induced wheeze [82, 83]. Currently, respiratory infections caused by viruses are recognized as a major public health problem because of the huge burden on individual health and economies, causing millions of deaths worldwide each year. NF-*κ*B transcription mediates the production of cytokines and chemokines in response to Toll-like receptor (TLR3) recognition of intermediate dsRNA during viral infections. Notably, several *in vitro* revealed that resveratrol regulated Toll-like receptor 3 (TLR3) expression, inhibited the TRIF signaling pathway, and induced M2 receptor expression following RSV infection [84]. Additionally, resveratrol appears to block the activities of the TIR-domain-containing adapter-inducing interferon-*β* (TRIF) complex, suggesting that resveratrol would also inhibit NF-*κ*B transcription induced by TRIF [85]. A review had summarized that resveratrol also reduced the activity of respiratory syncytial virus and inhibited the Toll/IL-1 receptor domain-containing adaptor inducing *β* interferon (TRIF) expression through upregulating sterile *α* and armadillo motif protein (SARM) [86]. Moreover, resveratrol, as a SIRT-1 agonist, inhibits the replication of RSV in human bronchial epithelial cells and stimulates the secretion of higher levels of TNF-*α*, thereby promoting cell death. In addition, resveratrol also promotes cellular defense systems and apoptosis, which means it is possible to promote RSV clearance in the body more quickly [87].

#### 4.2.2. Zika Virus

Zika virus (ZIKV) is a single-stranded RNA virus belonging to the *Flavivirus* genus in the *Flaviviridae* family. ZIKV remains an important cause of congenital microcephaly in a population where ZIKV has adapted to a nexus involving the Aedes mosquitoes and humans [88]. ZIKV infection has also been associated with an increased incidence of Guillain-Barré syndrome, which is an autoimmune neurological complication characterized by muscle weakness, motor dysfunction, and paralysis in some cases [89]. ZIKV has emerged as a pathogen of major health concern all over the world [90]. Resveratrol inhibits the replication of ZIKV in a dose-dependent manner. When the infected cells were treated with 80 *μ*M resveratrol, the virus titer and viral mRNA copy number were decreased by 90%, implying a possibility that resveratrol also interferes with ZIKV binding [91]. Notably, a study proved the protective effects of resveratrol on ZIKV-infected human RPE cells. Also, a study reported that resveratrol had a high affinity for two enzymes of the rate-limiting steps of pyrimidine and purine biosynthesis and viral polymerase [92]. Currently, the inhibition of resveratrol on the NS3 helicase of Zika virus has been demonstrated. Resveratrol stabilized the P-loop and blocked the RNA-binding pocket for 200 ns, and resveratrol binding significantly reduces ATP hydrolysis activity [93]. Resveratrol was able to interfere both in the early stages of the viral infectious cycle and in the late stages [94].

#### 4.2.3. Severe Acute Respiratory Syndrome Coronavirus 2

Coronavirus disease 2019 (COVID-19) is caused by a novel coronavirus known as Severe Acute Respiratory Syndrome Coronavirus 2 (SARS-CoV-2). It is an enveloped, nonsegmented, and positive sense RNA virus that is included in the sarbecovirus, ortho corona virinae subfamily which is broadly distributed in humans and other mammals [95]. Symptoms of COVID-19 are variable, but the most common are fever, cough, breathing difficulties, and loss of smell and taste. Most people affected by COVID-19 have mild to moderate symptoms and recover without special treatment [96]. COVID-19 is also a systemic inflammatory vascular disease, evident by the increased concentrations of proinflammatory cytokines in severe cases. The research suggested that resveratrol significantly suppresses cyclooxygenase-1 (COX-1), a key enzyme in the catalytic production of prostaglandins, which are key inflammatory mediators [97, 98]. In addition, resveratrol appears to sufficiently suppress expressions of these interleukins. Interestingly, The NF-*κ*B and Nrf2 signaling pathways play a significant role in cytokine storms and oxidative stress, which are the hallmarks of COVID-19 [99, 100]. There are also studies evidencing that resveratrol toward two key targets involved in SARS-CoV-2 viral infection—Spike viral protein and ACE2 host receptor—was investigated by molecular docking simulations [101]. Meanwhile, network pharmacology reveals that the shared targets between resveratrol and SARS-CoV-2 mainly involved the IL-7 signaling pathway, NF-*κ*B signaling pathway, and TNF signaling pathway [102]. In the clinical trial, participants were randomly assigned to receive either placebo or resveratrol, and no clinically significant adverse events were found as evaluated by the number of emergency department visits and incidence of pneumonia and pulmonary embolism [103]. In *in vitro* study, resveratrol significantly inhibited the replication of SARS-CoV-2 with an EC_50_ of 4.48 *μ*M [104]. Overall, these data support the potential utility of resveratrol on SARS-CoV-2 infection.

#### 4.2.4. Other RNA Viruses

Enterovirus 71 (EV71) is characterized by a single-stranded positive RNA genome, belonging to the genus *Enterovirus* within the family *Picornaviridae*. EV71 usually infects infants and young children under the age of 5 years with symptoms such as fever, blisters, and rashes on the skin. Hand-foot-and-mouth disease (HFMD) is caused by EV71 and is a critical public health threat, especially in the Asia-Pacific region [105]. Infection with this virus may lead to acute central nervous system complications, including meningitis, encephalitis, poliomyelitis-like paralysis, neurogenic pulmonary edema, and even death [106]. Studies suggested resveratrol has strong antiviral activity against EV71. By blocking the IKKs/NF-*κ*B signaling pathway, EV71 replication and cytokine secretion in RD cells were inhibited [107]. Interestingly, a study found that resveratrol-loaded nanoparticles remarkably reduced EV71-induced viral replication and inflammatory effects by inhibiting the oxidative stress-mediated ERS/autophagy signaling pathway [108]. Meanwhile, resveratrol also inhibited dengue virus by blocking translocation of high mobility group 1 (HMGB1), resulting in the retention of HMGB1 in the nucleus which continuously promotes ISG production [109]. It is reported that resveratrol strongly attenuated the replication of influenza virus in MDCK cells. This process involved the blockade of the nuclear-cytoplasmic translocation of viral ribonucleoproteins and decreased expressions of late viral proteins which is seemingly related to the inhibition of protein kinase C activity and its dependent pathways [110].

## 5. Antiviral Mechanisms of Resveratrol

The antiviral effects of resveratrol are well demonstrated. Currently, studies on the antiviral mechanism of resveratrol mainly focus on two points.

On the one hand, resveratrol possesses the ability to activate the host's immune defenses, turning on a complex network of bodies to fight or eliminate incoming viruses. NF-*κ*B belongs to a family of inducible nuclear transcription factors, which regulates a wide array of genes involved in various processes of antiviral activity, inflammatory, and immune response [48]. Meanwhile, it is noteworthy that Toll-like receptors (TLRs) are located upstream of the NF-*κ*B signaling pathway and are responsible for induction of antiviral innate immune responses by recognizing virus infection, which leads to the production of proinflammatory cytokines, chemokines, and interferons [111]. Thus, the TLRs/NF-*κ*B pathway has been extensively studied during viral infection. Likewise, most research on antiviral drugs has focused on this pathway. Resveratrol regulated TLR3 expression, thus affecting the recruitment of downstream related factors and finally affecting the regulation process of related signal pathways. Moreover, resveratrol inhibits PRV by inhibiting the I*κ*B*α* degradation induced by PRV infection, thereby inhibiting the activation of the NF-*κ*B cell signaling pathway, and subsequently inhibiting the transcription of viral genes, protein and DNA synthesis, and virion production [76]. Thus, the TLRs/NF-*κ*B signaling pathway plays a crucial role in the antiviral process of resveratrol.

**Figure 3:**
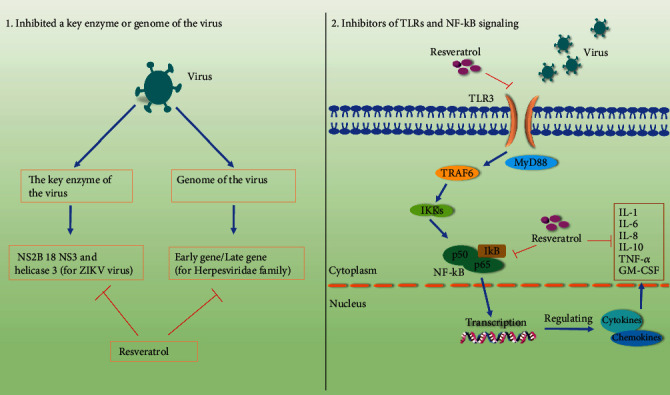
Schematic diagram of the possible mechanism by which resveratrol inhibits viruses.

On the other hand, resveratrol also has the ability to inhibit the production of virions by directly inhibiting the expression of key viral genes or by binding to key enzymes and proteins that disrupt the viral replication cycle. In this case, resveratrol could possess antiviral activities against ZIKV NS2B 18 NS3 and helicase 3 [91]. Overall, the antiviral mechanisms of resveratrol are diverse. We have summarized many studies and found that most of them focus on the effects of resveratrol on host signal pathways and key genes and proteins of viruses (Figure 3).

## 6. Summary and Perspectives

Active studies and many published articles have shed light on resveratrol's potential role in the treatment of a variety of diseases, particularly anti-inflammation and antivirus. In this review, we tried to summarize the anti-inflammatory and antiviral mechanisms of resveratrol. After reviewing many literatures, we concluded that the anti-inflammatory and antiviral effects of resveratrol involve the NF-*κ*B signaling pathway. When inflammation occurs, resveratrol can regulate the cascade reaction of NF-*κ*B signals, blocking the secretion of inflammatory factors, reducing the occurrence of inflammation, and enabling host cells to develop immune resistance to eliminate the virus. Therefore, the NF-*κ*B signaling pathway is the key way for resveratrol to exert anti-inflammatory and antiviral effects.

Therefore, future research should pay attention to two aspects: first, the anti-inflammatory and antiviral mechanisms of resveratrol can be traced back to related signaling pathways or upstream of NF-*κ*B; second, resveratrol has limited clinical research data, so clinical trials can be strengthened to provide support for the application of resveratrol.

## Figures and Tables

**Table 1:** Plant origin of resveratrol.

Species	Plant of source	Concentration	Reference(s)
*Polygonum cuspidatum Sieb.et Zucc*	Giant knotweed	11.88 mg/g	[11, 12]
*Polygonum cuspidatum syn. Fallopia japonica*	Japanese knotweed	4.30 mg/g	[13]
*Vitis amurensis* cv. *Rupr.*	Grape, skins, and seed	0.1 mg/100 g; 2 mg/g	[14–16]
*Sophora tonkinensis Gagnep*	Peanuts	0.13 *μ*g/g	[17]
*Polygonum cuspidatum*	Itadori plants; Itadori tea	Stem 497 *μ*g/g; 974 *μ*g/100 mL	[18]
Vitis rotundifolia Michx.	Muscadine grape	36.85 *μ*g/g	[19]
*Vaccinium corymbosum* L	Highbush blueberry	140 pmol/g; 1 pmol/g	[20, 21]
